# Architecture Assessment of the Chilean Epidemiological Surveillance System for Notifiable Diseases (EPIVIGILA): Qualitative Study

**DOI:** 10.2196/34387

**Published:** 2023-07-07

**Authors:** Carla Taramasco, Carla Rimassa

**Affiliations:** 1 Universidad Andrés Bello Facultad de Ingeniería Viña del Mar Chile; 2 Escuela de Ingeniería Informática Facultad de Ingeniería Universidad de Valparaíso Valparaíso Chile; 3 Núcleo Milenio de Sociomedicina Santiago Chile; 4 Universidad de Valparaíso Facultad de Medicina, Escuela de Fonoaudiología, Campus San Felipe Centro Interdisciplinario de Investigación en Salud Territorial San Felipe Chile

**Keywords:** eHealth, surveillance, mandatory reporting of infectious diseases, public health, data mining

## Abstract

**Background:**

To fulfill their epidemiological vigilance function, authorities require valid, complete, timely, precise, and reliable information. Advancements in new technologies have facilitated public health control through vigilance systems for notifiable diseases; these systems can gather large numbers of simultaneous notifications, process a wide array of data, and deliver updated information in real time to relevant decision-makers. A large worldwide deployment of new information technologies was seen during the COVID-19 pandemic; these technologies proved to be efficient, resourceful tools . Platform developers should seek self-evaluation strategies to optimize functionality or improve the capacity of national vigilance systems. These tools exist in the Latin American region at various development stages, although publications reporting architectural characteristics of these tools are scarce. International publications are more abundant a nd serve as a basis for comparing the standards that need to be met.

**Objective:**

This study aimed to assess the architecture of the Chilean epidemiological surveillance system for notifiable diseases (EPIVIGILA), as compared to that of the international systems reported in scientific publications.

**Methods:**

A search for scientific publications was conducted to identify systematic reviews that documented the architectural characteristics of disease notification and vigilance systems. EPIVIGILA was compared to other systems from countries in Africa, the Americas, Asia, Europe, and Oceania.

**Results:**

The following aspects of the architecture were identified: (1) notification provenance, (2) minimum data set, (3) database users, and (4) data quality control. The notifying organizations, including hospitals, clinics, laboratories, and medical consultation offices, were similar among the 13 countries analyzed; this contrasted with Chile, where the reporting agent is the physician who can belong to an organization. The minimum data set include patient identification, disease data, and general codifications. EPIVIGILA includes all these elements, in addition to symptomatology, hospitalization data, type of medicine and treatment result, and laboratory test types. The database users or data analyzers include public health organizations, research organizations, epidemiological organizations, health organizations or departments, and the Centers for Disease Control and Prevention. Finally, for data quality control, the criteria most often used were completeness, consistency, validity, timeliness, accuracy, and competencies.

**Conclusions:**

An efficient notification and vigilance system must be capable of promptly identifying probable risks as well as incidence and prevalence of the diseases under surveillance. EPIVIGILA has been shown to comply with high quality and functionality standards, at the level of developed countries, by achieving total national coverage and by providing timely, trustworthy, and complete information at high-security levels, thus obtaining positive assessment from national and international authorities.

## Introduction

In 1988, epidemiological surveillance was defined as the “systematic collection, analysis and dissemination of health data for the planning, implementation and evaluation of public health programmes” [1]; this definition is still used currently and is shared by the Ministry of Health in Chile [2,3]. In the case of transmissible diseases, surveillance is vital for monitoring public health trends and disease outbreaks [4]. According to the Pan American Health Organization, the emergence of infectious diseases represents a threat, emphasizing the need to update the essential public health functions [5], which implies access to comprehensive quality services; health promotion and healthy behaviors; addressing the social determinants of health; monitoring and evaluation; surveillance, control, and risk management; research and knowledge management; development of human resources for health; medicines and other health technologies; health financing; policies, legislation, and regulatory frameworks; as well as social participation and social mobilization. To comply with the above, authorities require valid, complete, timely, precise, and reliable information [6]. In parallel, there is the option to use technological systems, which are more expeditious and provide real-time information compared to manual procedures [7]. This demands that vigilance systems possess mandatory characteristics, including rigorous analysis, flexibility or continuous adaptation capacity, and data precision [8].

In Chile, the notifiable disease (ND) norms [9] state that the notification must be issued by an accredited user (eg, a physician) using the form available in the integrated vigilance platform for notifiable diseases—EPIVIGILA by RAVENO (referred to as EPIVIGILA) [10]. In this long and narrow country with a centralized government, the first COVID-19 case was detected on March 3, 2020, and a general quarantine was implemented on March 16, 2020. Thus, from the beginning, the EPIVIGILA platform was expected to show high accuracy, process a large number of daily notifications, and generate a robust database, all while facing a pandemic phenomenon that impacted different countries at the same time. This sanitary scenario witnessed a worldwide deployment of new information technologies, which proved to be efficient helping tools for public health control [11-13].

It is expected that platform developers should seek self-evaluation strategies to optimize functionality or improve the capacity of national ND vigilance systems. In fact, the tools should be sufficiently flexible to satisfy the authorities’ requirements amidst the turmoil of emergencies. Once these challenges are overcome, it is inspiring to learn about and incorporate the experiences of other systems developed in similar contexts. However, there are few scientific publications at the Latin American level that describe these systems’ characteristics. Although many countries have these tools, they are at different stages of development. For example, SINAN (Brazil) reports the lack of an integrated data system that is adjusted to the speed of disease propagation, sufficiently flexible to include new diseases, with the capability to communicate, make data available, and obtain application programming interface access to the notifications system. This is necessary to build tables and automated reports to minimize delay in the temporal and spatial tracking of notified and confirmed cases [14].

Regarding SNVS. 2.0 (Argentina), a failing issue is that in many provinces, the system coexists with other local systems, resulting in duplication inefficiencies and a lack of communication between them. This situation not only impacts data quality but also makes it evident that the SNVS is not tailored to the provincial management needs [15].

Regarding SIVIGILA (Colombia), Tuesca et al [16] highlight the need to strengthen the uniformity between reported data from the territorial institutions and mention that electronic transfer exhibits technological limitations in data processing due to slipups that cause fragmentation of the recorded information (inconsistencies and information gaps) provided to the health authorities [17].

In SIVE (Ecuador), decisions are based on a single vigilance subsystem called SIVE-Alerta; therefore, to ensure rigorous vigilance, it is necessary to strengthen and integrate the country’s subsystems, so as to allow for obtaining information from first-level health care establishments, including hospitals, private laboratories, and the community, among others [18].

Health surveillance has been approached in different areas; however, there are few publications that account for the architecture and functionality of national surveillance systems [19-21]. There are scientific publications at the international level that demonstrate the functions and components of national vigilance systems. Systematic reviews are especially useful in this context due to their comprehensive nature; they document diverse elements of vigilance systems in several countries, carrying out an analysis and comparison of the characteristics and attributes of those tools. The purpose of this work is to assess the architecture of the EPIVIGILA system in Chile.

## Methods

A search was conducted for scientific publications corresponding to systematic reviews that documented and compared the architecture characteristics of national disease notification and vigilance systems. Only one publication fulfilling this criterion was found [22]. The purpose of our study is to present the assessment of the EPIVIGILA system architecture, as compared to the architecture of the international systems reported in scientific publications.

This study reports and compares vigilance systems in Africa, America, Asia, Europe, and Oceania. The results present a comparison between the Chilean vigilance system for notifiable diseases, EPIVIGILA, and the reported systems, considering the following aspects of the architecture: (1) notification provenance, (2) minimum data set provided, (3) database users, and (4) data quality control.

## Results

Regarding the provenance of disease notifications, the 13 countries analyzed, namely Germany, Australia, Canada, China, South Korea, United States, New Zealand, the Netherlands, Sweden, South Africa, United Kingdom, and Taiwan, with the exception of Sri Lanka, share a common pattern, where the notifying organizations are hospitals, clinics, laboratories, and medical consultation offices. Some other documented entities as sources of notifications are health care centers, nursing homes, schools, jails, primary care units, and blood banks, among others.

**Figure 1:**
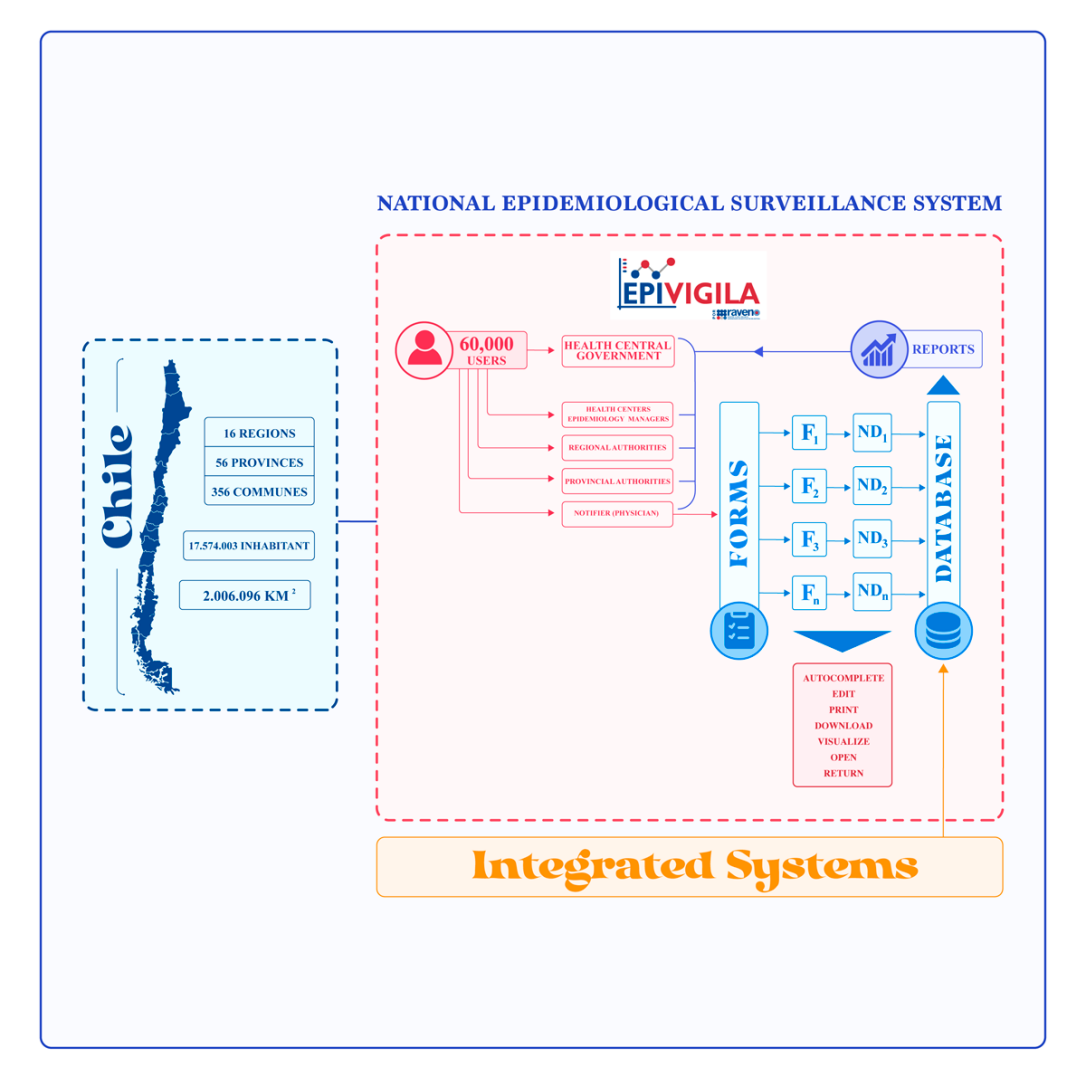
Architecture and functioning context of the the Chilean epidemiological surveillance system for notifiable diseases (EPIVIGILA).

In Chile, the reporting agent is not an entity or organization but a physician (according to the national norm) [9,10] who can be affiliated with a hospital, clinic, laboratory, or medical consultation. Therefore, this is a direct first-source report. The notifying person, wherever geographically situated, can enter the EPIVIGILA system and report the case. The only requirement is to have an internet connection. If the physician is accredited to work in Chile, they are automatically enrolled in the system using their Tax Number. This number, together with a unique log-in credential, allows access to the platform. The architecture and functioning context of the platform are shown in Figure 1.

Figure 1 shows that upon entering EPIVIGILA system, the notifying person is presented with different form options based on the specific disease. This modular approach enables the initial storage of data categorized by pathology. These data are then added to the final database, which includes all the NDs registered in the platform. Besides, EPIVIGILA is integrated with other systems, such as hospitals’ follow-up systems, sampling systems, the national immunization system, national civil records, national health insurance, and the health superintendence.

Regarding the minimum data set reported by vigilance systems in the compared countries, Haghiri et al [22] noted that, after conducting a systematic review, they identified clinical and nonclinical information encompassing a total of 77 elements to be included in the systems.

These elements can be grouped into 3 categories as follows: identification of the patient (eg, demographic, social, and economic antecedents), disease data (eg, diagnosis, code, agent, certainty level, reports periods, and contagion site), and general codifications (eg, the notifying agent code and the form code). EPIVIGILA includes all the elements reported in the compared countries; it additionally includes symptomatology, hospitalization data, type of medicine, treatment result, and laboratory test types. Specifically, during the COVID-19 pandemic, the system expanded to include a total of 150 fields; this allowed the generation of reports that were made available to the general community [23].

Table 1 shows a list of organizations with the types of data use (analysis or decision-making) in the epidemiologic vigilance systems across the countries compared.

**Table 1:** Users and types of data use (“A” representing analysis and “D” representing decisions) in the epidemiological vigilance systems of the countries compared.

Organization	United States	Australia	Canada	Sweden	Germany	Taiwan	China	South Korea	Netherlands	United Kingdom	Sri Lanka	New Zealand	South Africa	Chile (EPIVIGILA^a^)
Epidemiological organizations	A, D	A		A	A		D				A		A	A, D
Research organizations	A	A	A	A, D	A, D						A		A	A
Health organizations or departments	A, D	A, D		D	A	A				A, D		D	A	A
Centers for Disease Control and Prevention	A, D	D	D	A, D			A	D	A, D	D		D	D	A, D
Public health agencies	A, D	A	A-D	A			D	A	A, D	A, D	A	A	A	A, D
Government organizations or Ministry of Health	A	A, D	A	D			D					D		A, D
Health care teams		D	A	A		D				A	D	A		A, D
Laboratories		D	A, D			D	D							
Community health centers													D	
World Health Organization													D	
National health insurance													D	

^a^EPIVIGILA: the Chilean epidemiological surveillance system for notifiable diseases.

Table 1 shows a trend among the countries compared regarding the entities responsible for data analysis (data analyzers). Those analyzers include public health agencies, research organizations, epidemiological organizations, health organizations or departments, and the Centers for Disease Control and Prevention (CDC). On the other hand, the organizations that prevail in decision-making include CDC, public health organizations, health organizations or departments, health care teams, and laboratories.

The EPIVIGILA data analyzers include research organizations and health organizations or departments. In decision-making, alongside analysis, we identified epidemiological organizations, CDC, public health agencies, government organizations, or health care team, who mainly make decisions concerning aspects within their areas and scopes.

Regarding data quality control criteria in the ND system of the countries compared, it was reported that those most often used include completeness, consistency, validity, timeliness, accuracy, and competencies, although completeness, accuracy, and timeliness were considered to be the most important data quality criteria.

In Chile, EPIVIGILA operates national ND data integrated into a single system with high levels of granularity. This fine granularity regarding data details is important for public health vigilance because more analytical possibilities are available at a higher level of detail, allowing the authorities to visualize or identify the effects of the implemented strategies. According to the assessments of the national [24] and international (eg, World Health Organization) authorities [25], EPIVIGILA has high levels of compliance and performance. Its efficacy can be ascertained by examining the percentage of access errors, which revealed a rate of 0.18% of yearly errors for the year 2021, indicating an average availability of 99.82% during the past year [26]. Additionally, some data protection elements along the EPIVIGILA platform processing chain must be highlighted. Access is limited to users with unique and encrypted codes and passwords; there are different user roles that allow access to information based on profile types. Moreover, the data are also supervised by the platform’s management and development team [27] and central authorities (eg, the sanitary and Department of State authorities) and are subject to periodic security audit. Furthermore, the system is hosted on Amazon, which adheres to strict security rules for infrastructure.

## Discussion

### Principal Findings

By comparing the EPIVIGILA system architecture with the architecture of the international systems reported in scientific publications, we can identify 4 key elements. First, notification provenance differs in Chile compared to the 13 countries analyzed because the notifying agent is the physician and not the organization [9,10]. Second, the minimum data in surveillance systems, in general, include patient identification, disease data, and general codifications; EPIVIGILA complements this information by incorporating additional information, such as symptomatology, hospitalization data, type of medicine and treatment results, as well as laboratory test types. Third, EPIVIGILA users (analysts and decisions-makers) are similar to those mentioned in the reported developed countries, although at this point the delivery of information could be extended to other actors (eg, universities, schools, and municipalities) to attain integrated prevention strategies (primary, secondary, and tertiary); this is not a deficiency of the EPIVIGILA system, as it is related to the decisions made by the tool’s managers [2,3,6]. Fourth, regarding data quality, there is evidence of adequate performance, mainly measured in terms of integrity, exactness, and promptness [24,25].

Therefore, if a comparison between EPIVIGILA and those systems is sought, the minimum requirement would be to meet most of the requirements evidenced in the systems of developed countries. As we can see, the characteristics are similar or show minor differences. ND systems are oriented toward preventing the propagation of diseases or their effects, including epidemics and deaths. Therefore, an efficient notification and vigilance system must be capable of identifying probable risks as well as the incidence and prevalence of the diseases being tracked in the shortest possible time frame. This drives EPIVIGILA to incorporate an extended data set to improve clinical decision-making.

### Conclusions

A persistent challenge for disease vigilance system managers is to establish a network that strengthens these tools, both nationally and internationally, through the formation of alliances to exchange experience, which could optimize the integrated vigilance system for the benefit of the local and worldwide populations. EPIVIGILA has demonstrated compliance with high quality and functionality standards, positioning itself on par with systems used in developed countries. It has achieved total national coverage, delivering complete, timely, reliable information under high security measures. EPIVIGILA has obtained positive evaluations from both national and international authorities amid the challenging development of the pandemic.

## Abbreviations

CDC: Centers for Disease Control and Prevention
ND: notifiable disease
